# Supplementary figures and images for: Genome sequence of the ornamental plant Digitalis purpurea reveals the molecular basis of flower color and morphology variation
Source: BMC Genomics. 2026 May 1;27:432. doi: 10.1186/s12864-026-12889-3 (PMC13134276; doi:10.1186/s12864-026-12889-3)

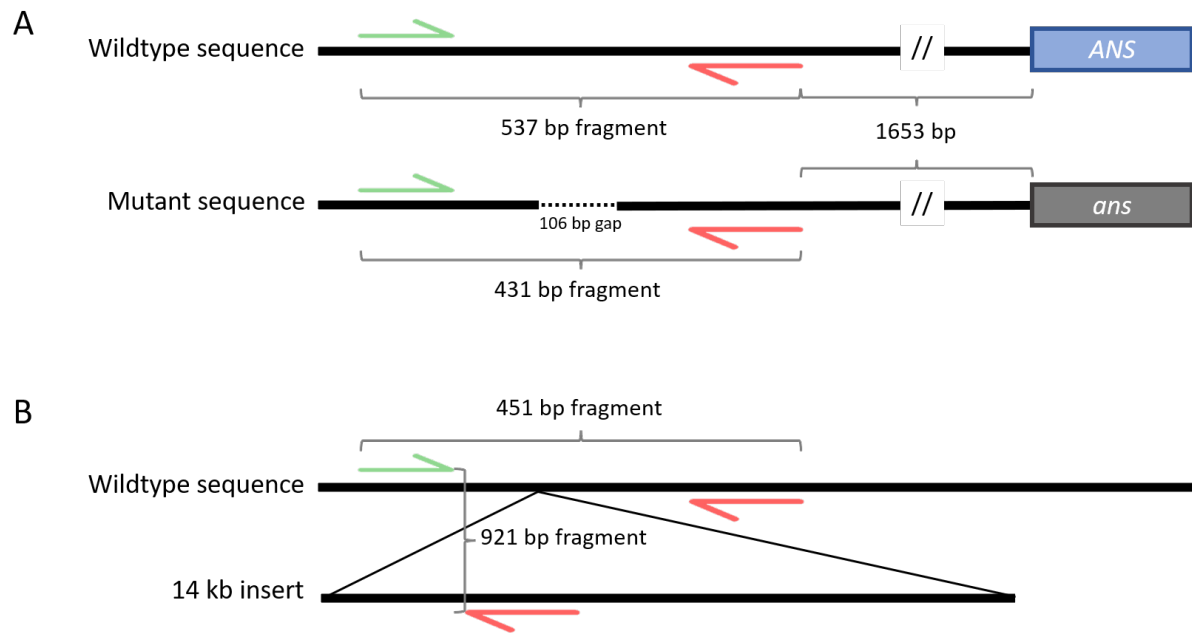

Design of the genotyping PCRs for the *DpANS* (A) and *DpTFL1/CEN* locus (B)

Supplement: Supplementary file 2 — Additional file 2: Documentation of all parameters and input files used for the annotation of the flavonoid biosynthesis genes with KIPEs3. [file 12864_2026_12889_MOESM2_ESM.pdf]

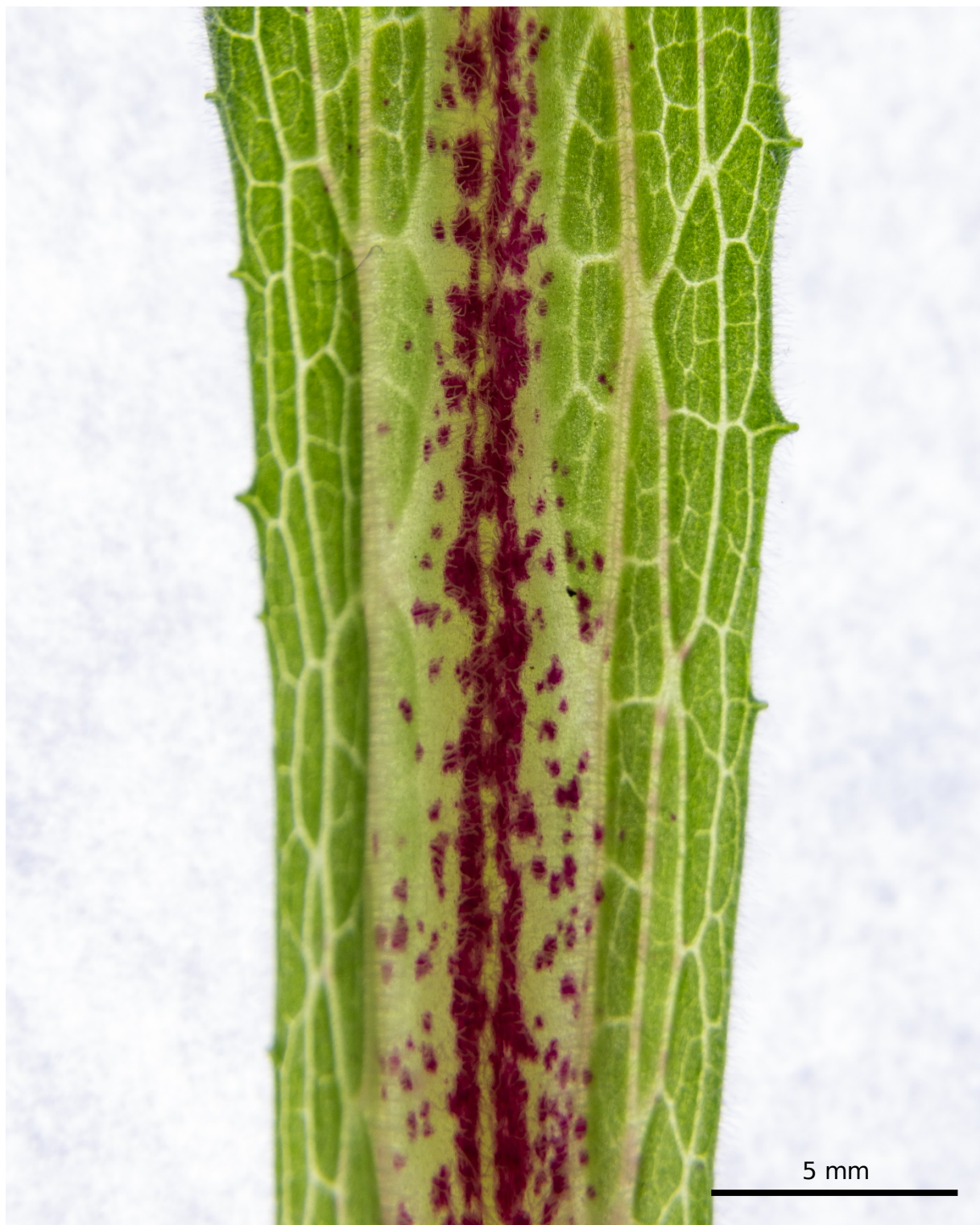

*Digitalis purpurea* leaf showing red pigmentation at the petiole.

Supplement: Supplementary file 18 — Additional file 18: Microsynteny plot showing collinear relationships between the contig harboring the ANS gene and a detected syntenic region. [file 12864_2026_12889_MOESM18_ESM.pdf]
