# Supplementary material for: Genome sequence of the ornamental plant Digitalis purpurea reveals the molecular basis of flower color and morphology variation
Source: BMC Genomics. 2026 May 1;27:432. doi: 10.1186/s12864-026-12889-3 (PMC13134276; doi:10.1186/s12864-026-12889-3)
Supplement: Supplementary file 8 — Additional file 8: D. purpurea plants showing magenta colored petioles. [file 12864_2026_12889_MOESM8_ESM.pdf]

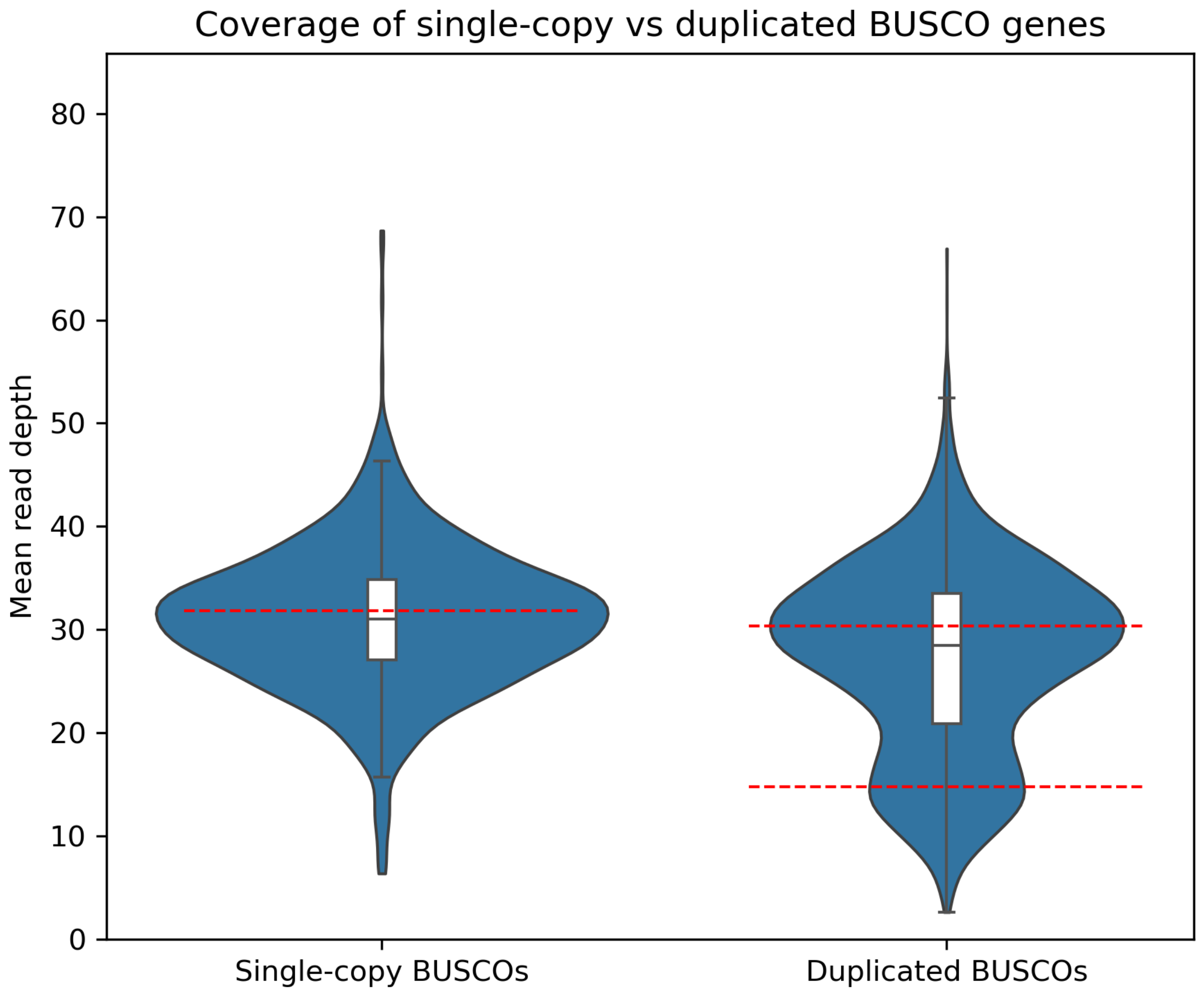

Fig. S1: Violin plots showing the distribution of mean read depth for single-copy and duplicated BUSCO genes. Red dashed horizontal lines denote coverage peaks identified by kernel density-based peak detection. Embedded box plots indicate median and interquartile range (IQR) with whiskers extending to 1.5x IQR.

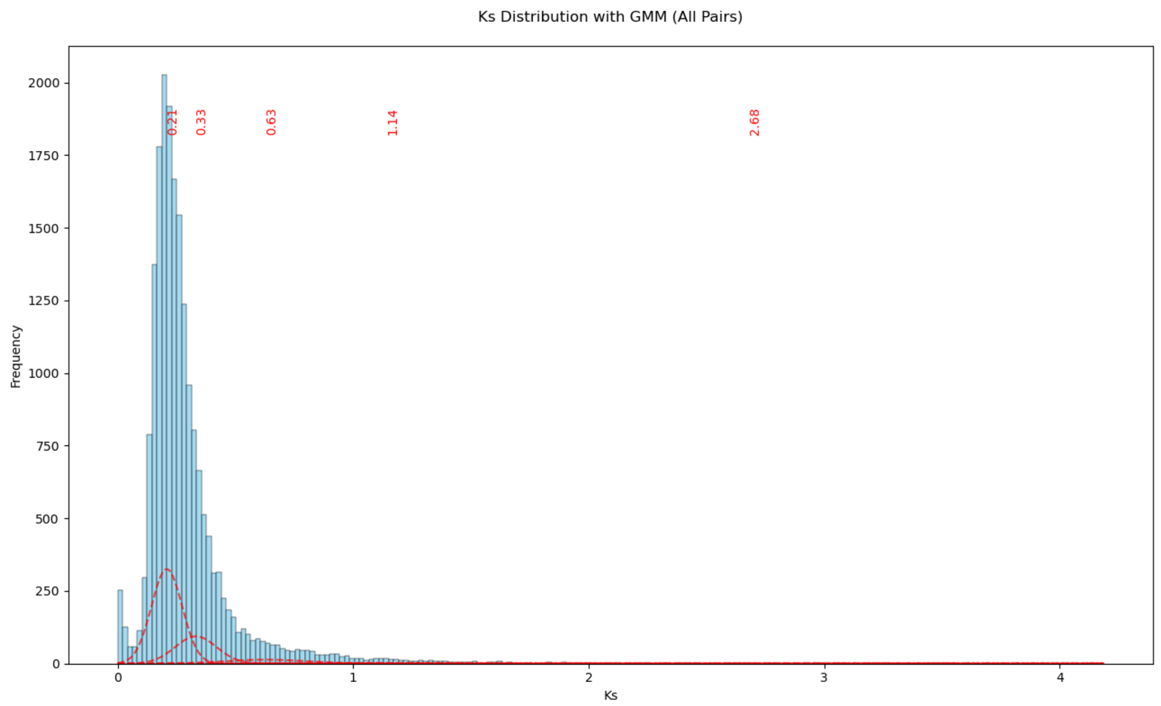

Fig. S2: Ks distribution across all syntenic gene pairs. Red dashed curves represent the individual components of a Gaussian mixture model (5 components) fitted to the Ks distribution, with red labels indicating the inferred Ks peak positions.

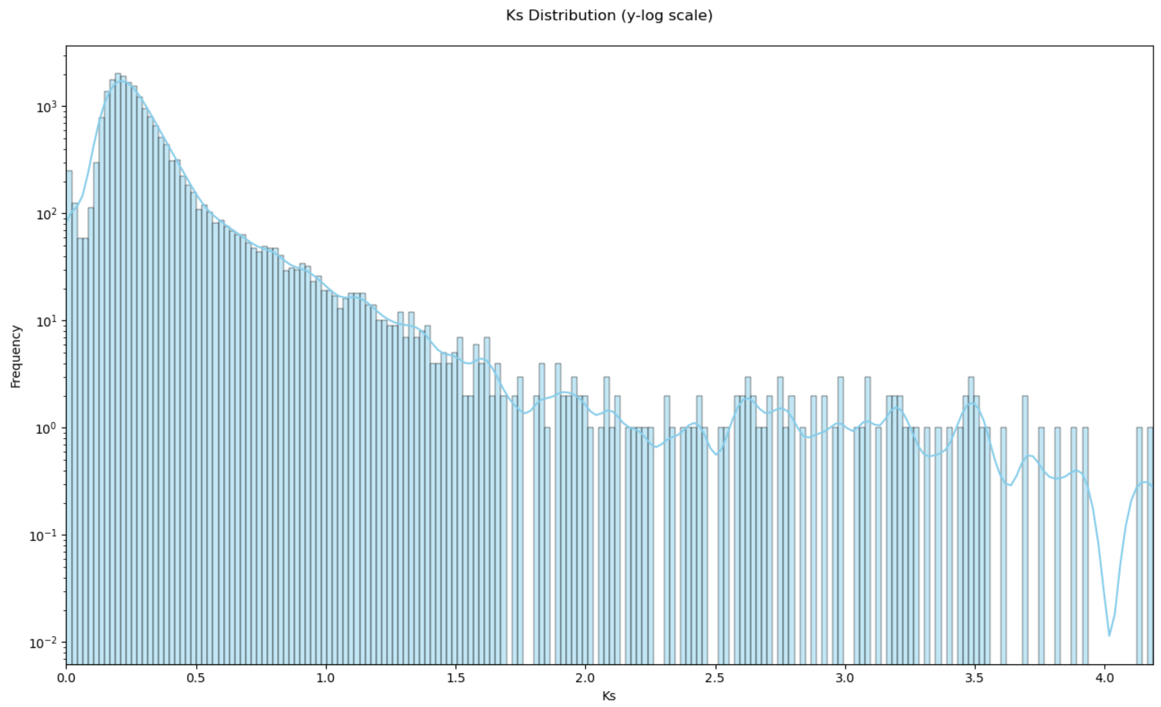

Fig. S3: Ks distribution across all syntenic gene pairs. Y-axis is displayed with log scale for visualization of lower occurrences
