# Supplementary material for: Genome sequence of the ornamental plant Digitalis purpurea reveals the molecular basis of flower color and morphology variation
Source: BMC Genomics. 2026 May 1;27:432. doi: 10.1186/s12864-026-12889-3 (PMC13134276; doi:10.1186/s12864-026-12889-3)
Supplement: Supplementary file 9 — Additional file 9: Coverage plots of single copy and duplicated BUSCO genes; Ks plot of all syntenic gene pairs and Ks plot of all syntenic gene pairs in y log scale. [file 12864_2026_12889_MOESM9_ESM.pdf]

TabS1: PCR cycle program for the *DpTFL1/CEN* mutant. For the touchdown PCR program, the annealing temperature was decreased by 1 degree each cycle until the terminal temperature was reached. Number of cycles was 45.

Step	Wildtype reaction		Mutant reaction	
	Time	Temperature	Time	Temperature
Initial Denaturation	5 min	95 °C	5 min	95 °C
Denaturation	1 min	95 °C	1 min	95 °C
Annealing	1 min	65 °C -> 59 °C	1 min	65 °C -> 59 °C
Extension	00:40 min	68 °C	1:05 min	68 °C
Primers	JH1	JH2	JH2	JH6

JH1 (wildtype reverse): 5’ AGGGTCTGTCATGATCTG 3’

JH2 (forward): 5’ TGTTCAATCCTCTTCATCTC 3’

JH6: (mutant reverse) 5’ TGTACGCGAATGAAGC 3’

TabS2: PCR cycle program for the *DpANS* mutant. Differently sized fragments will be obtained for wildtype and mutant target sequences, respectively. Number of cycles was 45.

Step	Time	Temperature
Initial Denaturation	5 min	95 °C
Denaturation	1 min	95 °C
Annealing	1 min	59 °C
Extension	00:45 min	68 °C

JH28: 5’ CTATAGTCGGGTCACATACGC 3’

JH29: 5’ CCTTTGAACATGGTGTCATAACC3’
