# Supplementary material for: Genome sequence of the ornamental plant Digitalis purpurea reveals the molecular basis of flower color and morphology variation
Source: BMC Genomics. 2026 May 1;27:432. doi: 10.1186/s12864-026-12889-3 (PMC13134276; doi:10.1186/s12864-026-12889-3)
Supplement: Supplementary file 12 — Additional file 12: Summary file output by EDTA. [file 12864_2026_12889_MOESM12_ESM.pdf]

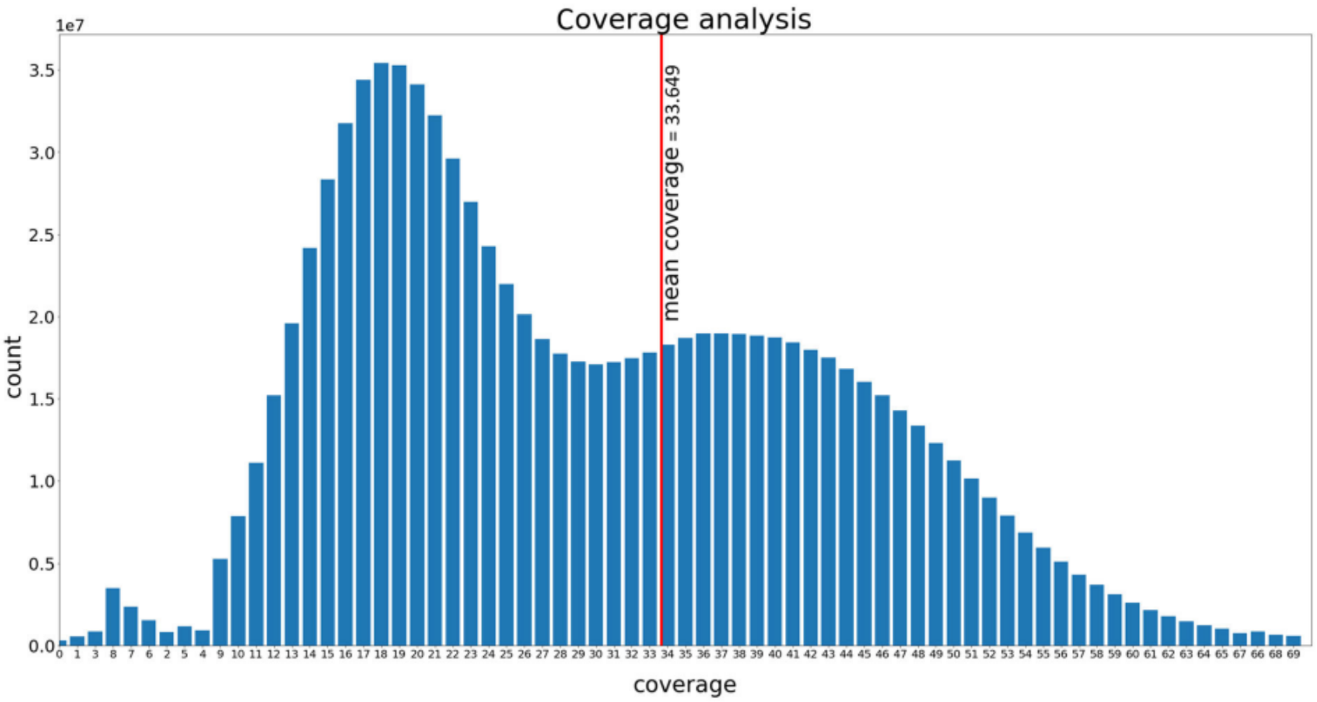

Coverage histogram of the DR1_v1 assembly. The coverage depth is displayed on the x-axis and the number of positions with a certain coverage is displayed on the y-axis. The mean coverage is depicted by the red line. The x-axis was cut at 69 for clarity.
