# Supplementary material for: Genome sequence of the ornamental plant Digitalis purpurea reveals the molecular basis of flower color and morphology variation
Source: BMC Genomics. 2026 May 1;27:432. doi: 10.1186/s12864-026-12889-3 (PMC13134276; doi:10.1186/s12864-026-12889-3)
Supplement: Supplementary file 14 — Additional file 14:Expression plot showing the activity of anthocyanin biosynthesis associated genes across different samples. [file 12864_2026_12889_MOESM14_ESM.pdf]

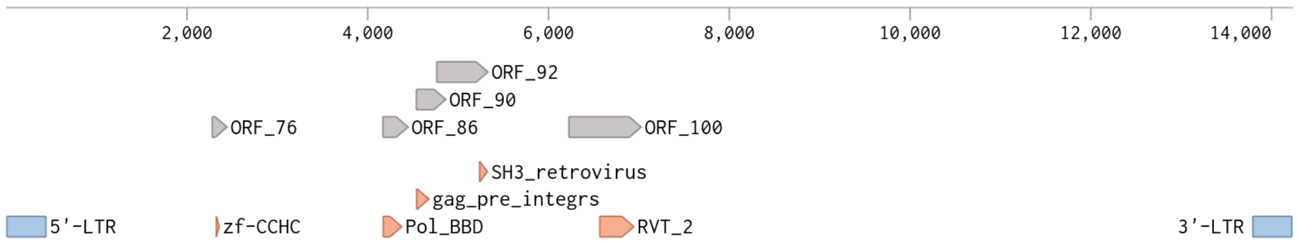

Elements on the putative TE inserted sequence in the *ans* gene. Blue: Flanking LTRs, Orange: Pfam hmmer motifs found on ORFs derived from the sequence. Gray: ORF for which the hmmer motivs were found. Sequence length is 14238 bp. ORF: Open reading frame, LTR: Long terminal repeat, zf-CCHC: zinc knuckle, Pol_BBD: Pol polyprotein, beta-barrel domain, gag_pre_integrs: GAG-pre-integrase, SH3_retrovirus: retroviral SH3-like fold, RVT_2: reverse transcriptase. Image was created with benchling.com
