# Supplementary material for: Genome sequence of the ornamental plant Digitalis purpurea reveals the molecular basis of flower color and morphology variation
Source: BMC Genomics. 2026 May 1;27:432. doi: 10.1186/s12864-026-12889-3 (PMC13134276; doi:10.1186/s12864-026-12889-3)
Supplement: Supplementary file 15 — Additional file 15: Documentation of the genotyping results of a D. purpurea population at the loci ANS and TFL1/CEN and and the corresponding phenotypes as well as the genotypes of the four sequenced individuals. [file 12864_2026_12889_MOESM15_ESM.pdf]

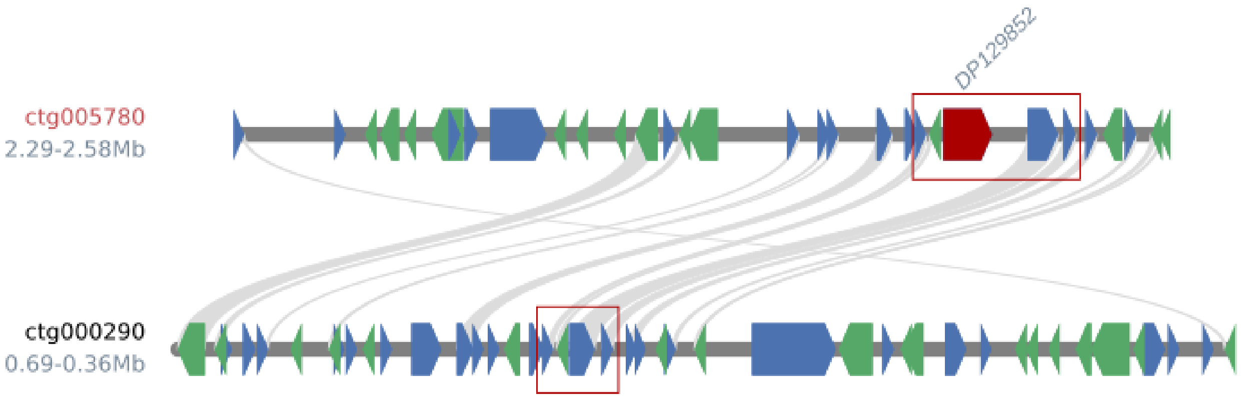

Microsynteny plot showing collinear relationships between the contig harbouring the *ANS* gene (highlighted in red) and a detected syntenic region (contig ctg000290). Red boxes highlight syntenic blocks comprising genes adjacent to ANS. No *ANS* paralog could be detected within the syntenic region on the paralogous contig.
