# Supplementary material for: Genome sequence of the ornamental plant Digitalis purpurea reveals the molecular basis of flower color and morphology variation
Source: BMC Genomics. 2026 May 1;27:432. doi: 10.1186/s12864-026-12889-3 (PMC13134276; doi:10.1186/s12864-026-12889-3)
Supplement: Supplementary file 17 — Additional file 17: Single LAST hit produced by searching the Assembly against the contig harboring the ANS gene. [file 12864_2026_12889_MOESM17_ESM.pdf]

Table S1: Genotyping results at the *ANS* locus of 61 plants with their respective phenotype. A: Wildtype *ANS*, a: Mutant *ans*.

Phenotype \ Genotype	A/A	A/a	a/a
Purple	10	26	2
White	0	2	21

Table S2: Genotyping results at the *TFL1/CEN* locus of plants with their respective phenotype. T: Wildtype *TFL1/CEN*, t: Mutant *tfl1/cen.*

Phenotype \ Genotype	T/T	T/t	t/t
Wildtype	4	13	0
Terminal flower	0	1	6

Table S3: Genotypes of the sequenced individuals, derived from read mappings. A: Wildtype *ANS*, a: Mutant *ans*, T: Wildtype *TFL1/CEN*, t: Mutant *tfl1/cen.*

ID \ Locus	ANS	TFL1/CEN
DR1	A/a	T/T
DR2	A/a	T/t
DW1	a/a	T/T
DW2	a/a	t/t
