# Supplementary material for: Genome sequence of the ornamental plant Digitalis purpurea reveals the molecular basis of flower color and morphology variation
Source: BMC Genomics. 2026 May 1;27:432. doi: 10.1186/s12864-026-12889-3 (PMC13134276; doi:10.1186/s12864-026-12889-3)
Supplement: Supplementary file 19 — Additional file 19: Magenta ﬂower (left) and white ﬂower (right) under UV-illumination. The two upper petals were removed to open the ﬂower and expose the inside of the remaining ﬂower. [file 12864_2026_12889_MOESM19_ESM.pdf]

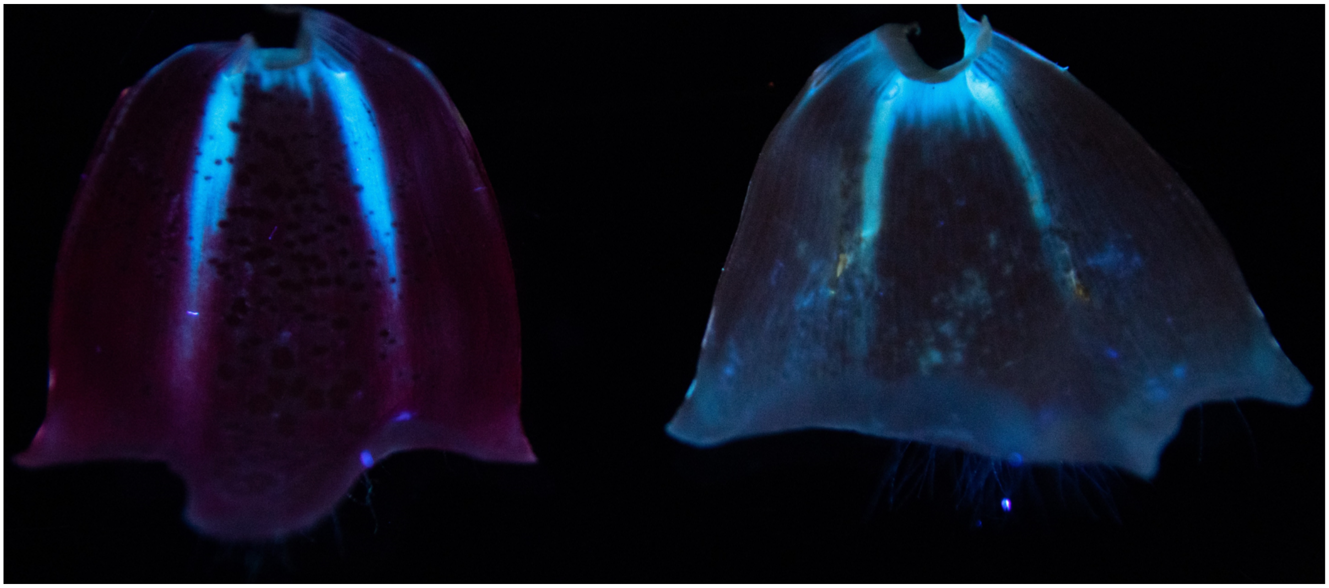

Magenta flower (left) and white flower (right) under UV-illumination. The two upper petals were removed to open the flower and expose the inside of the remaining flower.
